# On the Complex Flow Dynamics of Shear Thickening Fluids Entry Flows

**DOI:** 10.3390/mi15111281

**Published:** 2024-10-22

**Authors:** Miguel Montenegro, Francisco J. Galindo-Rosales

**Affiliations:** 1 Centro de Estudos de Fenómenos de Transporte (CEFT), Departmento de Engenharia Mecânica, Faculdade de Engenharia da Universidade do Porto, Rua Dr. Roberto Frias, 4200-465 Porto, Portugal; montenegro@fe.up.pt; 2 ALiCE—Laboratório Associado em Engenharia Química, Faculdade de Engenharia da Universidade do Porto, Rua Dr. Roberto Frias, 4200-465 Porto, Portugal; 3 Centro de Estudos de Fenómenos de Transporte (CEFT), Departamento de Engenharia Química, Faculdade de Engenharia da Universidade do Porto, Rua Dr. Roberto Frias, 4200-465 Porto, Portugal

**Keywords:** entry flow, continuous shear thickening fluids, CFD, microfluidics

## Abstract

Due to their nature, using shear thickening fluids (STFs) in engineering applications has sparked an interest in developing energy-dissipating systems, such as damping devices or shock absorbers. The Rheinforce technology allows the design of customized energy dissipative composites by embedding microfluidic channels filled with STFs in a scaffold material. One of the reasons for using microfluidic channels is that their shape can be numerically optimized to control pressure drop (also known as rectifiers); thus, by controlling the pressure drop, it is possible to control the energy dissipated by the viscous effect. Upon impact, the fluid is forced to flow through the microchannel, experiencing the typical entry flow until it reaches the fully developed flow. It is well-known for Newtonian fluid that the entrance flow is responsible for a non-negligible percentage of the total pressure drop in the fluid; therefore, an analysis of the fluid flow at the entry region for STFs is of paramount importance for an accurate design of the Rheinforce composites. This analysis has been numerically performed before for shear-thickening fluids modeled by a power-law model; however, as this constitutive model represents a continuously growing viscosity between end-viscosity plateau values, it is not representative of the characteristic viscosity curve of shear-thickening fluids, which typically exhibit a three-region shape (thinning-thickening-thinning). For the first time, the influence of these three regions on the entry flow on an axisymmetric pipe is analyzed. Two-dimensional numerical simulations have been performed for four STFs consisting of four dispersions of fumed silica nanoparticles in polypropylene glycol varying concentrations (7.5–20 wt%).

## 1. Introduction

Shear thickening fluids (STFs) are complex fluids, typically consisting of dense suspensions of solid particles dispersed in an inert carrier fluid, that exhibit an increase in viscosity under the application of a shear rate/stress over a critical value [1]. In other words, the stress required to shear an STF increases faster than linearly with the shear rate [2]. It is not an expected behavior in pure substances. Still, it can be observed in concentrated suspensions where the particles show no mutual attraction towards one another under no shearing flow [3]. Depending on the shape and size of particles, their concentration, the carrier fluid, etc., two kinds of shear thickening behaviors have been described in the literature, i.e., continuous shear thickening (CST) and discontinuous shear thickening (DST). In CST, the viscosity curve typically shows three regions: a first shear thinning followed by a shear thickening and, finally, a second shear thinning, which results from the shear-induced rearrangement of the particles [4]. At low shear rates, the particles may form layers and the viscosity reduces from the rest state (first shear thinning region); however, for shear rates over a critical value, the hydrodynamic forces disrupt this ordered state and randomly form hydrodynamic clusters of particles, resulting in an increase in the viscosity (shear thickening region) until reaching a maximum; if the shear rate is further increased, the hydroclusters become unstable, the shear forces break them down, and a new ordered state is reached, providing lower viscosity values (second shear thinning region) [5]. In DST, the viscosity curve exhibits a shear thickening region much steeper than in CST and no second shear thinning region; this is a consequence of the friction and jamming between particles [2].

**Figure 1:**
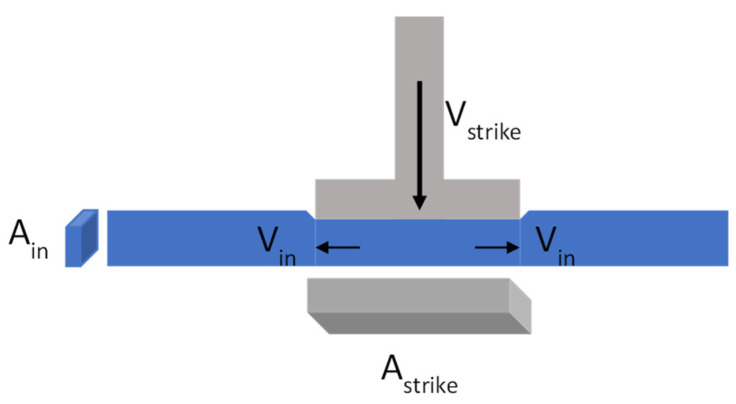
Sketch of the relationship between the velocity of the strike and the velocity of the fluid inside the microchannel.

Whereas CST has exhibited reversibility for stable, dense colloidal suspensions [6], the complete and spontaneous relaxation of the DST does not occur due to the partial retention of the frictional force chains [7,8]. For that reason, from the practical point of view, shear thickening fluids exhibiting continuous shear thickening with no hysteresis in the flow curve measurements are more interesting for the development of composites embedding shear thickening fluids [1,9,10,11,12,13,14,15,16,17]. Among these applications, we are particularly interested in Rheinforce technology, formerly known as CorkSTFluidics [18], which has been successfully applied in shin guards [19] and helmet liners [20]. This technology [21] consists of adding shear-thickening fluids to any resilient scaffold material by means of embedding microfluidic patterns; the right combination of the cushioning properties of the solid material, the energy-dissipating properties of the shear-thickening fluid, and the fluid–structure interaction results in a composite material that can be tuned to damp, potentially, any impact load. The use of a microfluidic network for embedding the STF into the resilient solid material introduces several major advantages with regards to other strategies, such as impregnation of fabrics or open-cell foams: (1) reduced amount of fluid [22], which is of paramount importance for applications in which lightweight is crucial; (2) enhanced rheological response of the STFs [23]; (3) the geometry of the microchannels can be numerically optimized to produce the desired fluid flow [24,25]. This latter feature is the key feature of this technology, as it has been proven to be possible to control very efficiently the pressure drop by means of designing the shape of the microchannels, also known as microfluidic rectifiers [26,27,28]. As A pressure drop is equivalent to the energy dissipation by unit volume of fluid, controlling the pressure drop in a microfluidic device is equal to controlling the amount of energy dissipated for a fixed amount of liquid. Upon the impact of the strike at a velocity $v_{strike}$, the fluid contained in the microfluidic channel under the impact zone will be forced to flow out of that region towards both sides of the microchannel with a velocity of $v_{in}=\frac{1}{2}\;v_{strike}\cdot \frac{A_{strike}}{A_{in}}$ [29] (Figure 1), which resembles the entry flow in a pipe.

The pressure drop experienced by the fluid multiplied by the flow rate determines the power of energy dissipated by the fluid due to viscous effects: $W=2\cdot \Delta p\cdot A_{in}\cdot v_{in}$. It is well known that the pressure drop experienced by a fluid along a horizontal pipe is the sum of the pressure drop at the entrance region and the pressure drop in the fully developed region [30]. Therefore, understanding the flow dynamics of shear thickening fluids in the entrance region is paramount for optimizing the design of the Rheinforce composites for anti-impact application.

The study of the entry flow in a pipe or a channel has been widely reported in the literature, both for Newtonian flows [31,32], as well as for viscoelastic [33] and inelastic non-Newtonian flows, such as in the works of Gupta [34], Chebbi [35], Poole and Ridley [36], Fernandes, et al. [37] and Lambride, et al. [38], where a power-law model fluid (Equation (1)) allows us to consider shear-thinning (n<1), Newtonian (n=1), and shear-thickening behaviors (n>1) just by playing with the values of the exponent parameter n:

(1) $$\eta =k\cdot {\dot{\gamma }}^{\left(n-1\right)},$$

where η is the fluid’s viscosity, k is the flow consistency index, n is the flow behavior index, and $\dot{\gamma }$ is the shear rate, which is defined as the second invariant of the rate of deformation tensor [39]. In these previous studies, the velocity profiles at different axial locations are reported and analyzed, and the influence of the power-law index on the development length of the velocity profiles in a 2D axisymmetric pipe is evaluated. In the work of Poole and Ridley [36], it was also reported the evolution of the velocity profiles along the development length region of the pipe when the fluid changes its rheological behavior: from shear thinning to Newtonian and to shear thickening. The power-law model (Equation (1)) is a monotonic function that either increases or decreases the viscosity values between two limiting plateaus at very low and very high viscosity values. Consequently, it is not able to predict the three characteristic regions typical of the CST behavior: Region I shear thinning at low shear rates, below the critical shear viscosity (${\dot{\gamma }}_{min}$); Region II shear thickening at intermediate shear rates (${\dot{\gamma }}_{min}\leq \dot{\gamma }\leq {\dot{\gamma }}_{max}$); and Region III ($\dot{\gamma }\geq {\dot{\gamma }}_{max}$) characterized by another shear thinning at high shear rates [40]. This study advances the state of the art by providing the first analysis of the effects of these three qs on the dynamics of the flow in the entry region, offering valuable insights for the improved design and modeling of energy dissipative composites with shear thickening fluids exhibiting CST behavior.

## 2. Materials and Methods

### 2.1. Governing Equations

Compared to 3D, 2D simulations reduce significantly the computational resources and time; moreover, modeling and solving axisymmetric problems in 2D is simpler and more straightforward. For the study of the laminar entry flow in a circular pipe, no limitations regarding deviations from the actual physical system are expected.

Consequently, the developed model consists of a 2D axisymmetric, incompressible, steady-state, laminar flow inside a microtube. The conservation equations calculated during the numerical procedure include the continuity (Equation (2)) and the momentum conservation equation (Equation (3)), in both the axial (z) and radial (r) directions:

(2) $$\nabla \cdot \left(\rho \vec{v}\right)=0,$$

(3) $$\nabla \cdot \left(\rho \vec{v}\vec{v}\right)=-\nabla p+\nabla \cdot \left(\overset{̿}{\tau }\right),$$

where ρ is the fluid’s density, $\vec{v}$ is the velocity, p is the static pressure, and $\overset{̿}{\tau }$ is the stress tensor which is given, in the case of a GNF constitutive model, by Equation (4):

(4) $$\overset{̿}{\tau }=\eta \left(\dot{\gamma }\right)\left[\left(\nabla \vec{v}+\nabla {\vec{v}}^{T}\right)\right],$$

being $\eta \left(\dot{\gamma }\right)$ a function showing the dependence of the dynamic viscosity with the shear rate. In this study, it was considered the GNF model proposed by Khandavalli, et al. [41] (Equation (5)):

(5) $$\eta \left(\dot{\gamma }\right)=\left\{\frac{\eta_{\infty }}{A}+\left(\eta_{0}-\frac{\eta_{\infty }}{A}\right)\cdot {\left[1+{\left(\lambda_{1}\cdot \dot{\gamma }\right)}^{B_{1}}\right]}^{\frac{n_{1}-1}{B_{1}}}\right\}\cdot \left\{1+A-A\cdot \left[1+{\left[{\left(\lambda_{2}\cdot \dot{\gamma }\right)}^{B_{2}}\right]}^{\frac{n_{2}-1}{B_{2}}}\right]\right\},$$

where $\eta_{0}$ is the zero-shear viscosity, $\eta_{\infty }$ represents the infinite-shear viscosity, A is a parameter that determines the extent of shear-thickening, $\lambda_{i}$ are time constants and, therefore, the inverse of the critical shear rates for thinning and thickening, $n_{i}$ represent the power-law exponents and $B_{i}$ are dimensionless transition parameters [41]. Equation (5) is a suitable GNF model for CST, as it is a smooth function of the viscosity (contemplating the three characteristic regions typically exhibited by shear thickening fluids: slight shear thinning at low shear rates, followed by a sharp viscosity increase over the critical shear rate and a subsequent pronounced shear thinning region at high shear rates [40]) that avoids discontinuities near the minimum and maximum values, unlike the piece-wise models proposed by Galindo-Rosales et al. [40,42]. Additionally, it has fewer parameters to fit the experimental data, which makes it more convenient for numerical simulations. The values of all these parameters for the four test fluids used in the study are presented in Table 1.

**Figure 2:**
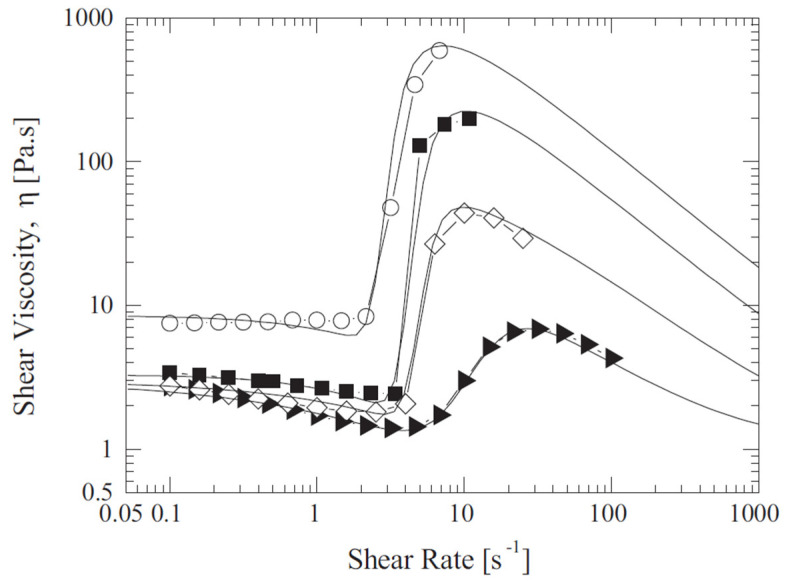
Steady shear viscosity curves for the four formulations as a function of shear rate. The data include different particle concentrations of (►) 7.5 wt%, (◇) 10 wt%, (■) 15 wt% and (○) 20%. Solid lines are fits of the high-rate-thinning model of Equation (5). Reprinted from [41], with permission from Elsevier.

Figure 2 presents the viscosity curves obtained with Equation (5) for each STF formulation, where the shear thickening region takes place between the shear rates of $1\leq \dot{\gamma }\leq 100$ s^−1^. The solid lines are fits of the high-rate-thinning model of Equation (5) to experimental steady shear rheology data for the four particle concentrations obtained by Khandavalli, Lee, Pasquali and Rothstein [41].

### 2.2. Geometry, Boundary Conditions, Mesh Analysis and Numerical Methods

The fluid domain consisted of a cylinder of length L=1 mm. Three different diameters were considered, i.e., $D=\left\{0.05,\;0.1,\;0.2\right\}$ mm. The axisymmetric nature of the flow allowed us to perform two-dimensional (2D) numerical simulations.

**Figure 3:**
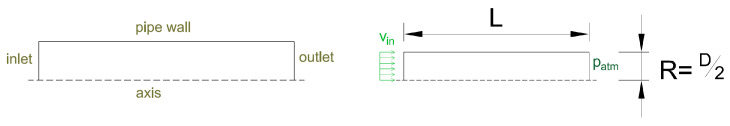
Sketch of the channel geometry and boundary conditions.

Non-slip condition, $v_{z}(r=R)=0$ was imposed on the pipe wall. A constant velocity profile is imposed at the inlet, $v_{z}\left(r\right)=v_{in}$. To cover the shear thickening region of the viscosity curve, the inlet velocity values were chosen between 0.1 mm/s and 1.5 mm/s. At the outlet, the fluid is discharged to atmospheric pressure, $p_{out}=p_{atm}.$ The channel geometry and the boundary conditions are summed in Figure 3.

**Figure 4:**
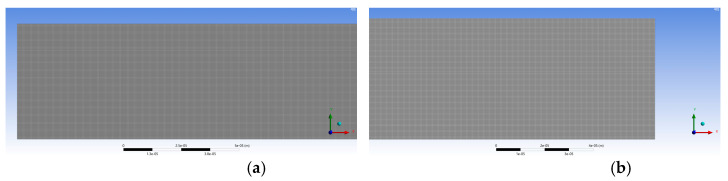
Mesh: (**a**) at the inlet region; (**b**) at the outlet region.

A size bias was used along the walls and at the pipe inlet for the mesh used in the present work (Figure 4). This is the region where the flow profile will develop and, therefore, the region of greater interest, hence the decision to refine this area of the tube. The convergence criteria adopted established that all residuals were below $1\times 10^{-9}$ and that at least 2000 iterations had been calculated. To guarantee the independence of the results from the mesh, a convergence study was carried out with three different meshes. The properties of each mesh are shown in Table 2. All the meshes present excellent cell quality regarding the skewness and the orthogonal quality, and the threshold aspect ratio of 1:5 is fulfilled [43].

**Figure 5:**
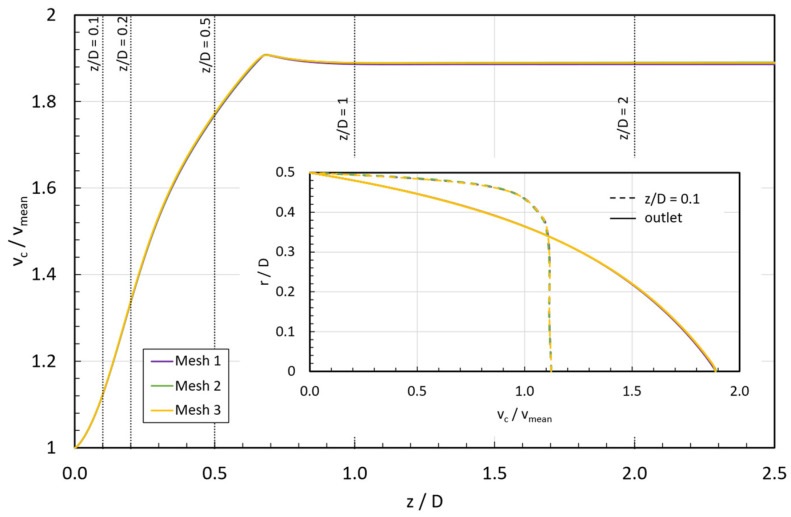
Comparison of the mesh influence on normalized axial velocities and velocity profiles for STF 4 at $v_{in}=0.25$ mm/s.

The convergence study was carried out for the fluid with the steepest growth of viscosity in the shear thickening region (STF 4) and an inlet velocity of 0.25 mm/s. Figure 5 illustrates the evolution of the normalized axial velocity $v_{c}/v_{in}$ along the tube; the inset graph shows the velocity profile at the normalized coordinate z/D=0.1 and the fully developed profile at the outlet obtained for the three meshes.

The velocity distribution and profiles along the pipe are consistent across the three meshes, ensuring the mesh independence of the numerical simulation. Additionally, the estimation of the uncertainty due to discretization in the simulations of the normalized axial velocity was also quantified using the Grid Convergence Method recommended in [44], and the results are reported in Table 3.

According to Table 3, the numerical uncertainty in the fine-grid solution for the normalized axial velocity at the selected normalized axial coordinates is always below 1%. Therefore, mesh 3 is chosen for the subsequent simulations because it balances computational efficiency and accuracy, especially near the critical zones (boundary wall and inlet region) where a finer mesh is needed.

The numerical simulations were performed using the commercial software FLUENT 2019 R2 distributed by ANSYS^®^ using the available flow models with a pressure-based solver and simulating the shear thickening effect by implementing the constitutive equation (Equation (5)) for the STFs through a user-defined function (UDF). For the numerical discretization of the governing equations presented in Section 2.1, the Green–Gauss Node-Based gradient evaluation is used. This discretization method computes the face value of a given variable through the arithmetic average of the nodal values on the face; this gradient scheme is more accurate than the cell-based gradient on irregular (skewed and distorted) meshes [43]. The coupling between the pressure and velocity fields is achieved through the segregated SIMPLE (Semi-Implicit Method for Pressure-Linked Equations) algorithm.

## 3. Results

### 3.1. Entrance Length

**Figure 6:**
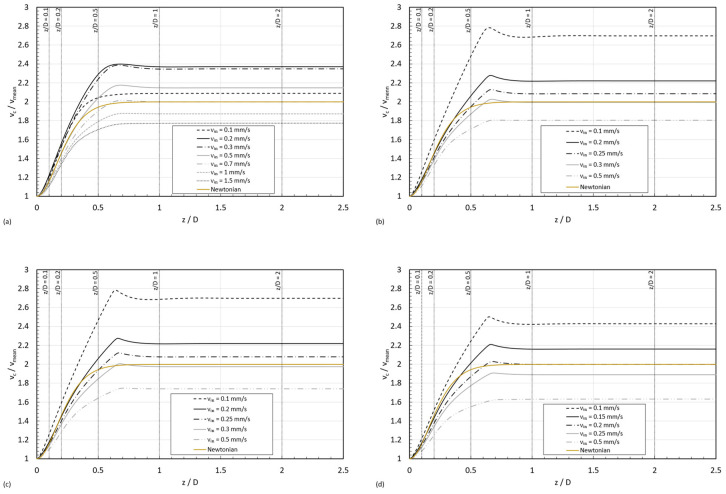
Normalized axial velocity for the four formulations of four shear thickening fluids with increasing concentration for increasing inlet velocities (**a**) STF 1, (**b**) STF 2, (**c**) STF 3, and (**d**) STF 4.

Figure 6 shows the normalized axial velocity, $v_{c}/\bar{v}$, along the normalized pipe length, z/D, for the four STF formulations. The presented velocity values are the maximum ones, calculated at the axis of the pipe. The results correspond to the inlet velocities simulations described in Section 2.2 in a pipe with the geometry L × D=1 × 0.1 mm. In each subfigure, it was also added the curve corresponding to a Newtonian fluid.

The STFs have different normalized axial velocity profiles from the Newtonian fluid, which has an unchanging curve for any inlet velocity. This difference is due to the shear thickening effect, which causes a local microstructure to form under shear. The inlet velocity affects how the STFs behave, changing the shear rate and the microstructure formation. Thus, for the same channel size and the same inlet flow rates, the normalized axial velocity curves vary depending on the viscosity curve of the STF; however, a general trend can be observed: it grows with the normalized position up to a maximum value, and then it slightly decreases until reaching the fully developed region. This phenomenon challenges the conventional definition of entry region for these fluids.

The entrance length, $L_{e}$, is the distance from where the fluid enters the pipe to where the boundary layer reaches the centerline and the velocity profile becomes fully developed and constant [45]. Lambride and co-workers [38] conducted a numerical study on the flow behavior of power-law fluids in pipes and channels. They computed the entrance length as a function of the transverse coordinate using a different definition based on the wall shear stress evolution. They found that the stress entrance length was lower than the conventional centerline entrance length for shear-thinning fluids (especially at low Reynolds numbers). However, for pipe flow, they showed that the usual definition of the development length was a good measure of flow development for power-law exponent values above 0.7, regardless of the Reynolds number. This indicated that the flow developed more slowly at the symmetry axis in shear-thickening fluids [38]. Based on their results, in this study we stick to the conventional centerline entrance length definition.

For Newtonian [31,32], viscoelastic [33], and inelastic non-Newtonian shear thinning fluids [34,35,36,37,38], the normalized axial velocity increases monotonically with the normalized z-position, so it is usually assumed that the velocity profile is fully developed when the centerline velocity, $v_{c}$, is at least 99% of the maximum velocity, $v_{max}$, which is equal to the exit velocity at the centerline, $v_{out}$. However, in most simulations, STFs show a peak in the centerline velocity before the profile is fully developed, so the criteria for estimating the entrance length in this work must account for this feature. Therefore, a different method was used to solve this problem: starting from the outlet and moving towards the inlet, if there is no peak, the entrance length is where the centerline velocity is lower than 99.9% of $v_{out}$; If there is a peak in the centerline velocity, the entrance length is where the centerline velocity is higher than 100.1% of $v_{out}$.

**Figure 7:**
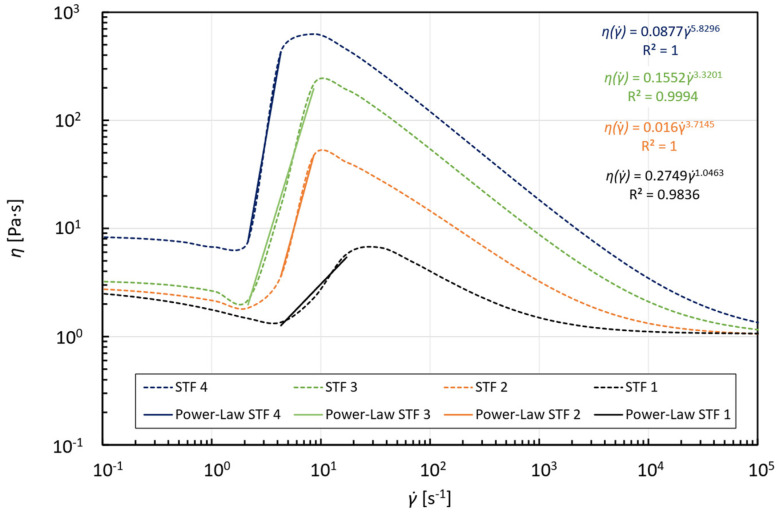
Shear-thickening region fitted to the power-law model (Equation (1)).

In creeping flow (Re≪1), the vast majority of research works reported that the entry length for Newtonian fluids follows Equation (6):

(6) $$\frac{L_{e}}{D}=C_{1}+C_{2}\cdot Re,$$

where $C_{1}$ is the asymptotic limit of the entrance length value when Re→0 [32]. For a better understanding of the values available in the literature for the coefficients in Equation (6), the reading of the works of Poole and Ridley [36], Li, et al. [46], and Ferreira, Sucena, Ferrás, Pinho and Afonso [32] is suggested. Regarding non-Newtonian fluids, the definition of the Reynolds number is not straightforward due to the dependence of the viscosity on the shear rate. The use of the Reynolds number developed by Metzner and Reed [47] (${Re}_{MR}=\frac{\rho {{\cdot v}_{mean}}^{2-n}{\cdot D}^{n}}{k}\cdot 8\cdot {\left(\frac{n}{6\cdot n+2}\right)}^{n}$, being $v_{mean}=v_{in}$ in this study) allows the development length at a high Reynolds number to collapse onto a single curve (Equation (6)) in which the coefficients are independent of the n index [36]. Since the model developed by Khandavalli, Lee, Pasquali and Rothstein [41] consists of a product of two Carreau–Yasuda models (one each for shear-thinning and shear-thickening regions, indicated by the subscripts 1 and 2, respectively), the shear thickening region of each fluid was approximated to a power trendline (Figure 7) in order to obtain both the flow consistency index k and the flow behavior index n for each formulation that allows the calculation of the aforementioned power-law Reynolds number.

**Figure 8:**
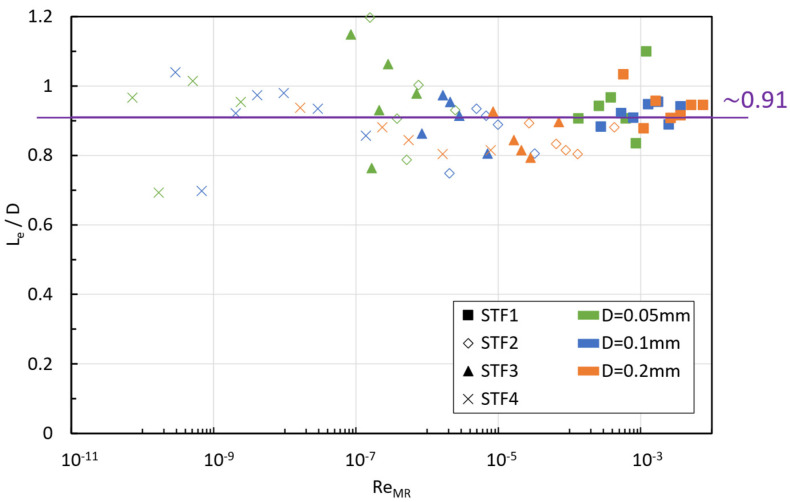
Entrance length vs. Reynolds number.

Figure 8 presents the normalized entrance lengths, $L_{e}/D$, as a function of ${Re}_{MR}$; it can be observed that the Reynolds numbers lay below $10^{-2}$, evidencing that the fluid is working in creeping flow and, consequently, at such a low Reynolds regime, the normalized entry length remains constant and independent of the n index. This result is consistent with the plots shown in Figure 6, as the axial centerline velocity profile seems to be constant for z/D>0.91.

### 3.2. Flow-Type Parameter

As mentioned above, within the entry region ($z<L_{e}$), depending on the inlet velocity and the rheological properties of the STF, the normalized axial velocity curves grows from 1 up to a maximum that can be larger than the value corresponding to the fully developed velocity. Poole and Ridley [36] observed a similar overshoot in the centerline velocity for power-law shear thickening fluids (n>1). However, the cause of this overshoot has never been analyzed in detail.

**Figure 9:**
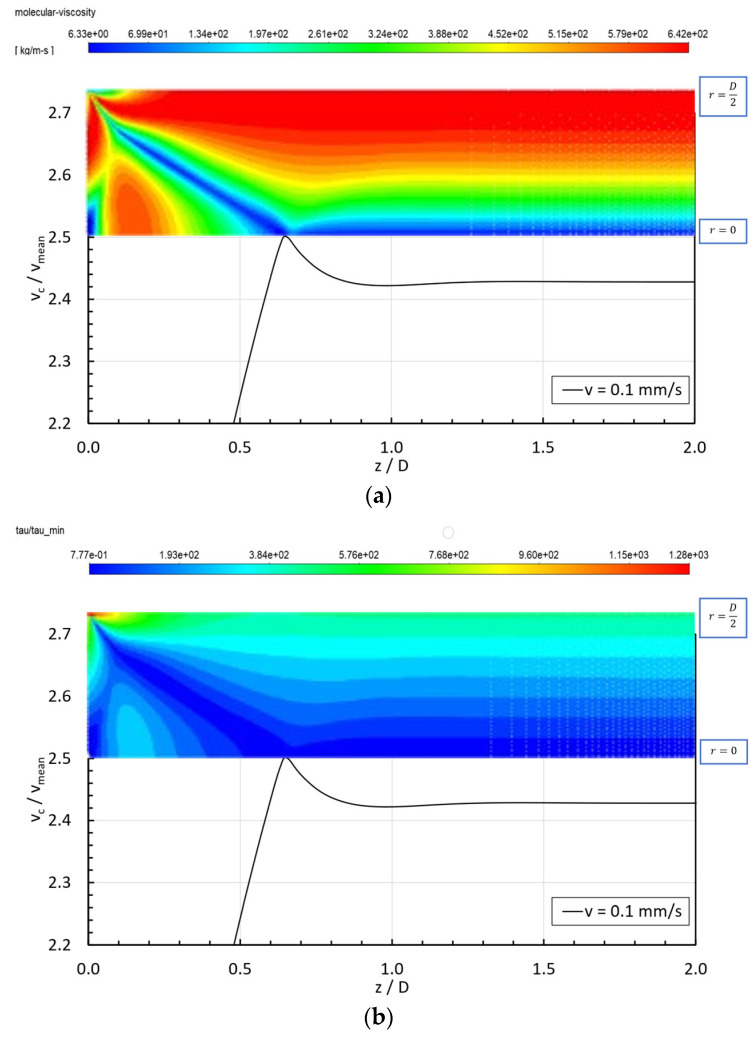

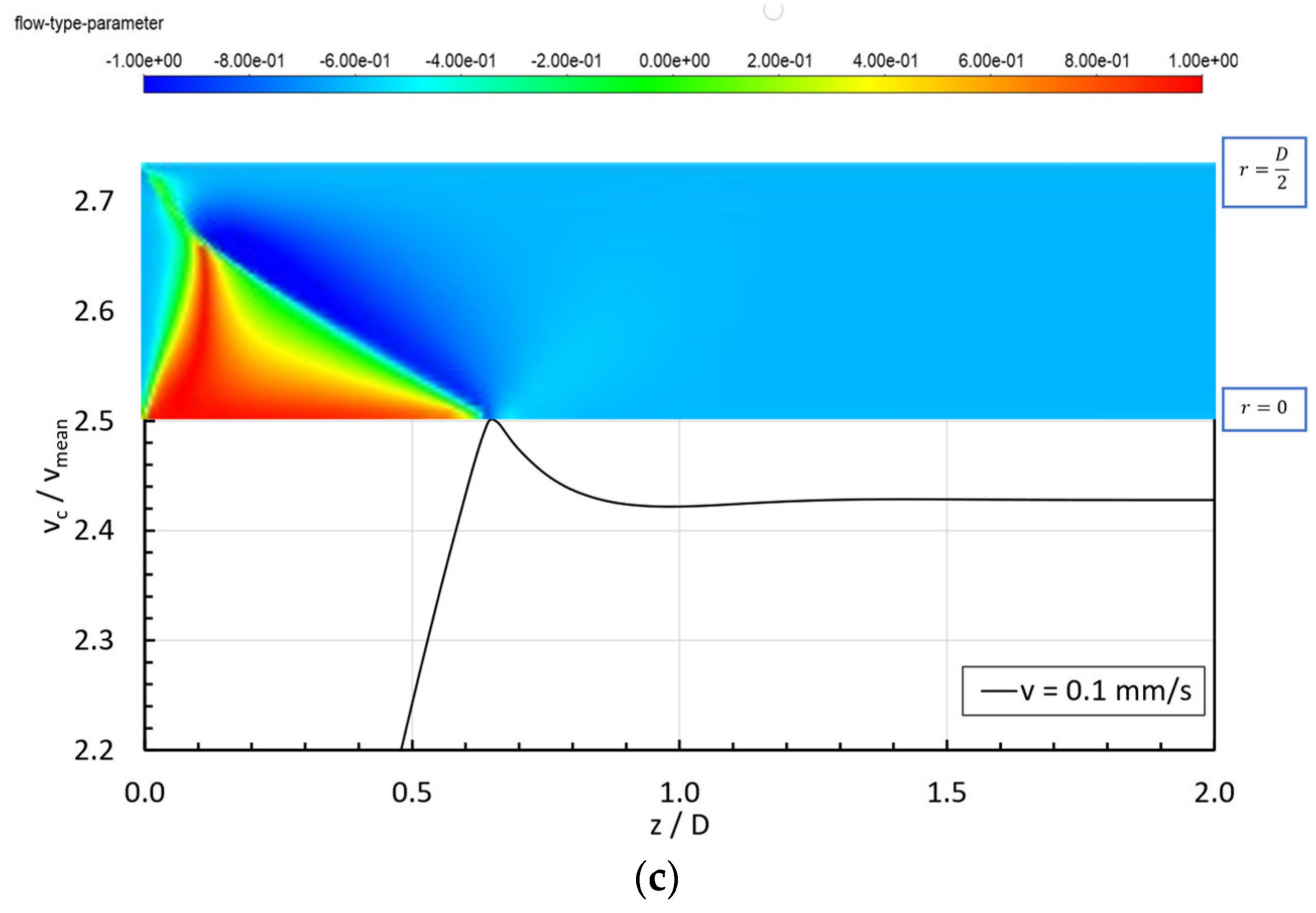
(**a**)—STF 4 viscosity along the pipe for $v_{in}=0.1$ mm/s and matching normalized axial velocity. (**b**)–STF 4 normalized shear stress along the pipe for $v_{in}=0.1$ mm/s and matching normalized axial velocity. (**c**)—STF 4 flow-type parameter along the pipe for $v_{in}=0.1$ mm/s and matching normalized axial velocity.

Figure 9a,b show the changes in the viscosity and stresses within the entry region of the STF 4 formulation for the case in which the inlet velocity is 0.1 mm/s. It is noticeable that the fluid adapts to the flow with a V-shaped peak appearing in the entry region, providing a minimum value of viscosity in every location at which the stresses are minimum, i.e., the critical shear stress ($\tau_{min}$) providing the onset of the shear thickening behavior (Figure 2). That viscosity and normalized shear stress ($\frac{\tau }{\tau_{min}}$) contour plots are a consequence of imposing a complex flow to the shear-thickening fluids, as in the centerline (r=0) the fluid is being undergone a pure elongational flow with $\dot{\varepsilon }=\frac{dv_{z}}{dz}$ and at the wall (r=D/2) the flow was simple shear with $\dot{\gamma }=\frac{dv_{r}}{dz}$. The fluid domain in between the centerline and the wall of the pipe will undergo a complex flow. The complexity of the flow is well represented by the flow-type parameter [48], defined by Equation (7):

(7) $$\xi =\frac{\left\|D\right\|-\left\|\Omega \right\|}{\left\|D\right\|+\left\|\Omega \right\|},$$

where ‖D‖ is the magnitude of the rate-of-deformation tensor and ‖Ω‖ is the magnitude of the vorticity tensor. Thus, when ξ=1, the region of the fluid domain is dominated by purely elongational flow; if ξ=0, the flow is dominated by simple shear; if ξ=−1, the flow approaches a solid-body rotation; finally, other portions show a combination of these [49]. Figure 9c) shows that, within the entry region, the viscosity is minimum when the flow approaches a solid-body rotation, and it is maximum in those locations where the shear or extensional flows are strong. The peak in the velocity profile appears when the local shear rate provides the minimum local viscosity in the surroundings of the centerline at the end of the entry length, where the solid rotation encounters the centerline; thus, the local viscosity is minimum, and the fluid is squeezed axially, providing the overshoot in the axial velocity at the centerline.

For the fully developed region ($z>L_{e}$), when the velocity profile does not depend on the z-position, the extensional flow disappears ($\dot{\varepsilon }=\frac{dv_{z}}{dz}$) and the fluid undergoes simple shear flow throughout the whole fluid domain [50].

**Figure 10:**
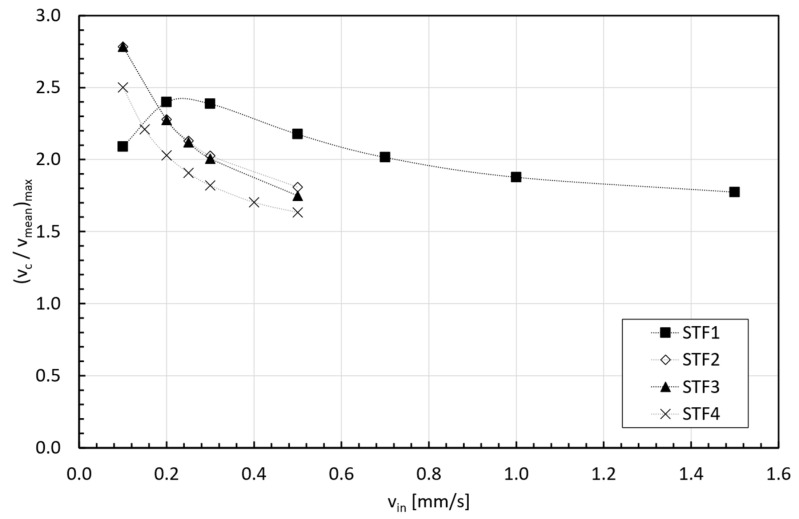
Evolution of peak normalized velocity with inlet velocity.

Further analyzing Figure 6, it can be observed that the peak value in the normalized axial velocity at the centerline also depended on the imposed inlet velocity and the viscosity curve of the fluid. Figure 10 shows that the milder shear thickening fluid (STF 1) exhibited a maximum in $\frac{v_{c}}{v_{mean}}$ increasing with v_in until reaching a maximum, and then it started decreasing below the Newtonian limit ($\frac{v_{c}}{v_{mean}}=2$). STF 2, STF 3, and STF 4 exhibited similar trends in the peak dependency with $v_{in}$, starting for the highest value in $\frac{v_{c}}{v_{mean}}$ and, subsequently, decreasing exponentially with $v_{in}$ again below the Newtonian limit. However, for STF 4, the fluid with the strongest shear thickening response, the starting peak value was lower than the one for STF 2 and STF 3.

This effect was not observed before for STFs because the considered power-law equation provides a viscosity that increases monotonically for n>1 [36]; however, the viscosity curves are not, exhibiting three regions (Shear thinning [Ⅰ]—Shear thickening [Ⅱ]—Shear thinning [III]) [40,42], as depicted in Figure 2. It is, therefore, paramount to analyze the shape of the velocity profiles in combination with the viscosity curves to fully understand the results shown in Figure 10.

### 3.3. Velocity Profiles

**Figure 11:**
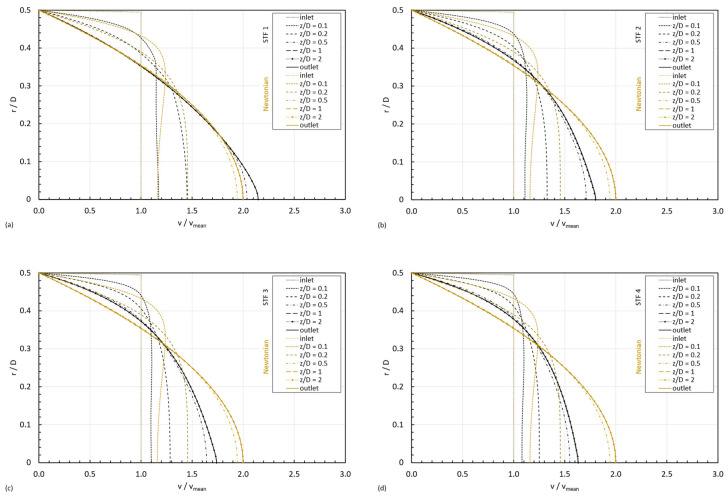
Velocity profiles for the four formulations of shear thickening fluids with increasing concentration in a D=0.1 mm pipe: (**a**) STF 1, (**b**) STF 2, (**c**) STF 3, (**d**) STF 4.

Figure 11 shows the velocity profiles for the four STF formulations with an inlet velocity of 0.5 mm/s at different locations in the z-direction, from the inlet to the fully developed region, compared to the Newtonian case. The remaining velocity profiles for the additional inlet velocities are shown in Appendix A.

It can be observed that, for the same inlet velocity, when the shear thinning behavior dominates next to the centerline, the maximum normalized velocity is smaller than the maximum normalized velocity developed by a Newtonian fluid. The contrary happens when the shear thickening behavior is triggered next to the centerline; the maximum normalized velocity for a shear thickening behavior is larger than that for a Newtonian fluid. These graphs are consistent with the results shown by Poole and Ridley [36] and Lambride, Syrakos and Georgiou [38]. The shape of the developed velocity profile is responsible for a gradient of shear rates in the radial direction that grows from a minimum at the centerline (${\dot{\gamma }}_{c}$) towards a maximum at the wall of the pipe (${\dot{\gamma }}_{w}$).

In the case of modeling the shear thickening behavior by a power-law model, as the curve viscosity monotonically increases from low to high viscosity values, there is a monotonically increasing viscosity from low to high values from the centerline to the wall. However, the viscosity model proposed by Khandavalli, Lee, Pasquali and Rothstein [41] is richer than the power-law model in the sense that it is able to cover the three typical regions in the shear thickening behavior; consequently, depending on the formulation of the fluid, the inlet velocity, and the dimensions of the pipe, different velocity profiles are developed, resulting in different values for ${\dot{\gamma }}_{c}$ and ${\dot{\gamma }}_{w}$ (${\dot{\gamma }}_{c}<{\dot{\gamma }}_{w}$), which may lead to six different cases depending on their respective location in the viscosity curve:

Case 1: both ${\dot{\gamma }}_{c}$ and ${\dot{\gamma }}_{w}$ are within Region I. The first shear thinning behavior dominates the whole fluid domain, and the viscosity decreases monotonically from the centerline towards the wall in the radial direction.
Case 2: ${\dot{\gamma }}_{c}$ belongs to Region I and ${\dot{\gamma }}_{w}$ is within Region II. The shear thinning behavior is dominating next to the centerline, and the shear thickening does it next to the wall; consequently, there is a non-monotonical variation in viscosities in the radial direction, and there will be a minimum in the viscosity at a certain distance from the centerline when the shear rate reaches the ${\dot{\gamma }}_{c}$ in the viscosity curve.
Case 3: ${\dot{\gamma }}_{c}$ belongs to Region I and ${\dot{\gamma }}_{w}$ is within Region III. The shear thinning behavior is dominating next to the centerline and next to the wall; however, the fact of reaching the two critical shear rates (${\dot{\gamma }}_{min}$ and ${\dot{\gamma }}_{max}$) in the viscosity curve results in a non-monotonical variation in viscosities in the radial direction. The viscosity will diminish from the centerline towards a minimum at a certain distance from the centerline; then it will increase until the maximum in shear rate, closer to the wall; and, finally, the viscosity will decrease from that maximum until reaching ${\dot{\gamma }}_{w}$ at the wall.
Case 4: both ${\dot{\gamma }}_{c}$ and ${\dot{\gamma }}_{w}$ are within Region II. The shear thickening behavior is dominating the whole fluid domain, and the viscosity increases monotonically from the centerline towards the wall in the radial direction.
Case 5: ${\dot{\gamma }}_{c}$ belongs to Region II and ${\dot{\gamma }}_{w}$ is within Region III. The shear thickening behavior is dominating next to the centerline, and the shear thinning does it next to the wall. Consequently, the viscosity will increase from the centerline to reach a maximum at a certain distance, and, from that position, it will decrease towards the wall of the pipe.
Case 6: both ${\dot{\gamma }}_{c}$ and ${\dot{\gamma }}_{w}$ belong to Region III. This scenario is similar to case 1 in the sense that the viscosity decreases radially from the centerline towards the wall of the pipe, but in this case, it follows the second shear thinning and not the first one in the viscosity curve.

The above discussion is valid only for axial positions beyond the entry length when the velocity profile reaches a fully developed shape and the flow type corresponds to simple shear. To observe which of the previously discussed scenarios is taking place in any of the different flow configurations considered in this study, it is preferable to normalize the shear rate for each formulation according to Equation (8):

(8) $$\dot{\gamma }*=\frac{\left(\dot{\gamma }-{\dot{\gamma }}_{min}\right)}{\left({\dot{\gamma }}_{max}-{\dot{\gamma }}_{min}\right)},$$

where ${\dot{\gamma }}_{min}$ is the minimum shear rate corresponding to the start of the shear thickening region and ${\dot{\gamma }}_{max}$ is the shear rate corresponding to maximum viscosity. Thus, $\dot{\gamma }*<0$ represents that Region I is excited, $0<\dot{\gamma }*<1$ indicates that Region II is triggered, and $\dot{\gamma }*>1$ shows that the fluid is working at Region III.

**Figure 12:**
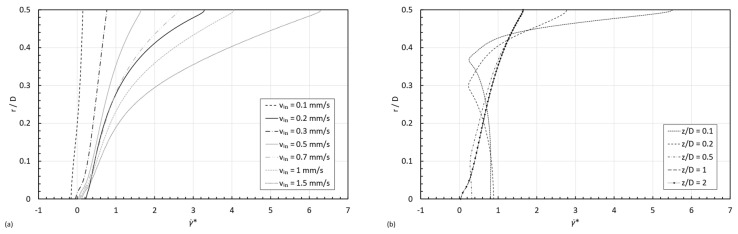
STF 1 normalized shear rate profile: (**a**) in the fully developed region for all inlet velocities and (**b**) at different z/D for $v_{in}=0.5$ mm/s.

Within the entry region, the situation is much complex due to the gradient in the flow-type from the centerline to the wall of the pipe, which is different at different locations in the axial direction. Figure 12 shows the normalized shear rate against the normalized radius for STF 1. In the fully developed region, depending on the inlet velocity, three different cases can be observed, i.e., cases 1, 2 and 5.

In the entry length, just for a given inlet velocity, depending on the position in the axial direction, the shear rate can be larger at the center than at the wall (${\dot{\gamma }}_{c}>{\dot{\gamma }}_{w}$), due to the extensional flow contribution at the centerline; consequently, the following cases arise:

Case 7: both ${\dot{\gamma }}_{c}$ and ${\dot{\gamma }}_{w}$ are within Region I. Since ${\dot{\gamma }}_{c}>{\dot{\gamma }}_{w}$, the viscosity increases from the centerline towards the wall. It is the reversed situation discussed in case 1.
Case 8: ${\dot{\gamma }}_{w}$ belongs to Region I and ${\dot{\gamma }}_{c}$ is within Region II. The shear thinning behavior is dominating next to the wall, and the shear thickening does it next to the centerline, resulting in the reverse situation described in case 2.
Case 9: ${\dot{\gamma }}_{w}$ belongs to Region I and ${\dot{\gamma }}_{c}$ is within Region III. The second shear thinning behavior is dominating next to the centerline, whereas the first shear thinning does it next to the wall, resulting in the reversed case 3.
Case 10: both ${\dot{\gamma }}_{c}$ and ${\dot{\gamma }}_{w}$ are within Region II. The shear thickening behavior is dominating the whole fluid domain, but because ${\dot{\gamma }}_{c}>{\dot{\gamma }}_{w}$, the viscosity decreases monotonically from the centerline towards the wall in the radial direction.
Case 11: ${\dot{\gamma }}_{w}$ belongs to Region II and ${\dot{\gamma }}_{c}$ is within Region III. The shear thickening behavior is dominating next to the wall, and the shear thinning does it next to the centerline. Consequently, the viscosity will increase from the centerline to reach a maximum at a certain distance, and, from that position, it will decrease towards the wall of the pipe.
Case 12: both ${\dot{\gamma }}_{c}$ and ${\dot{\gamma }}_{w}$ belong to Region III. This scenario is similar to case 7 in the sense that the viscosity increases radially from the centerline towards the wall of the pipe, but in this case, it follows the second shear thinning and not the first one in the viscosity curve.

**Figure 13:**
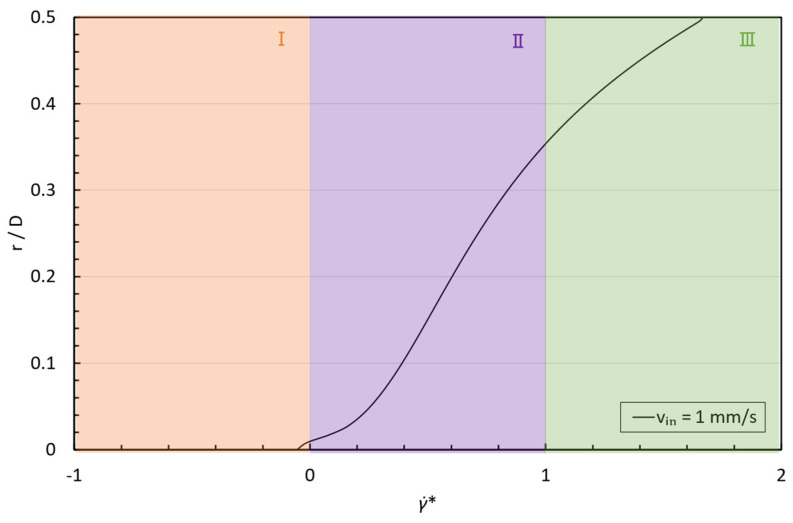
STF 1 normalized shear rate profiles at normalized coordinate z/D=2, in a D=0.2 mm pipe for $v_{in}=1$ mm/s. I, II and III represent each of the zones of the viscosity curve for a CST, according to Galindo-Rosales, et al. [40,42].

For the flow configurations considered in this study, we could observe the following cases: 1, 2, 5, 10, and 12, as shown in Figure 12. However, if we play with the dimensions of the pipe, other cases arise, like case 3, illustrated in Figure 13, where the STF 1 normalized shear rate profile at normalized coordinate z/D=2, in a D=0.2 mm pipe for an inlet velocity of 1 mm/s is shown.

**Figure A1:**
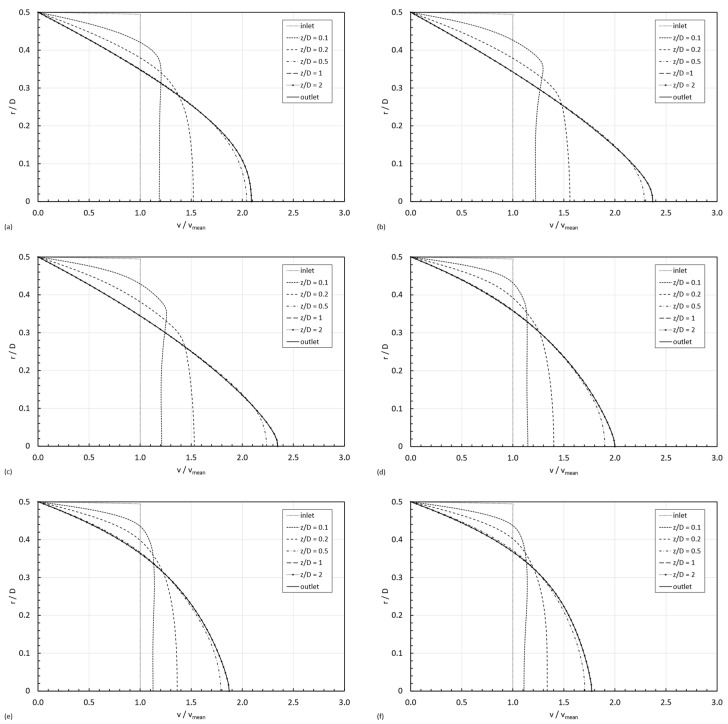
Velocity profiles for the STF 1 formulation in a D=0.1 mm pipe: $v_{in}=$ (**a**) 0.1 mm/s, (**b**) 0.2 mm/s, (**c**) 0.3 mm/s, (**d**) 0.7 mm/s, (**e**) 1 mm/s, and (**f**) 1.5 mm/s.

**Figure A2:**
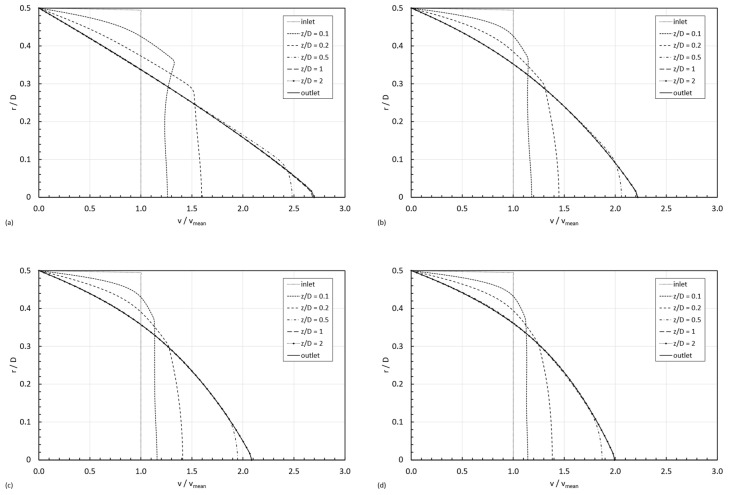
Velocity profiles for the STF 2 formulation in a D=0.1 mm pipe: $v_{in}=$ (**a**) 0.1 mm/s, (**b**) 0.2 mm/s, (**c**) 0.25 mm/s, and (**d**) 0.3 mm/s.

**Figure A3:**
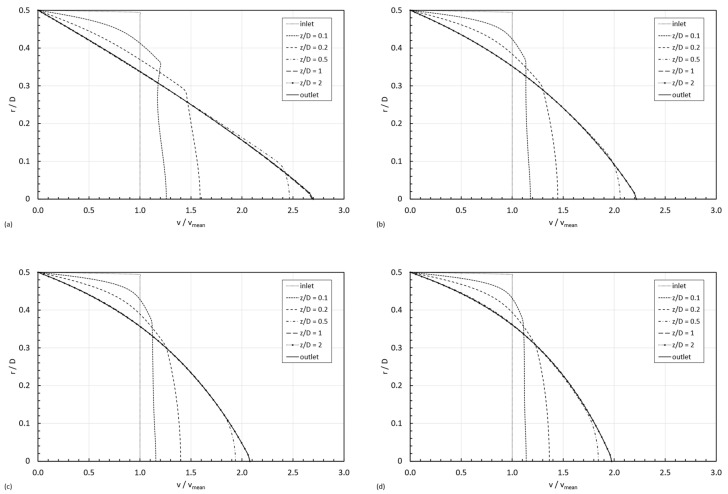
Velocity profiles for the STF 3 formulation in a D=0.1 mm pipe: $v_{in}=$ (**a**) 0.1 mm/s, (**b**) 0.2 mm/s, (**c**) 0.25 mm/s, and (**d**) 0.3 mm/s.

**Figure A4:**
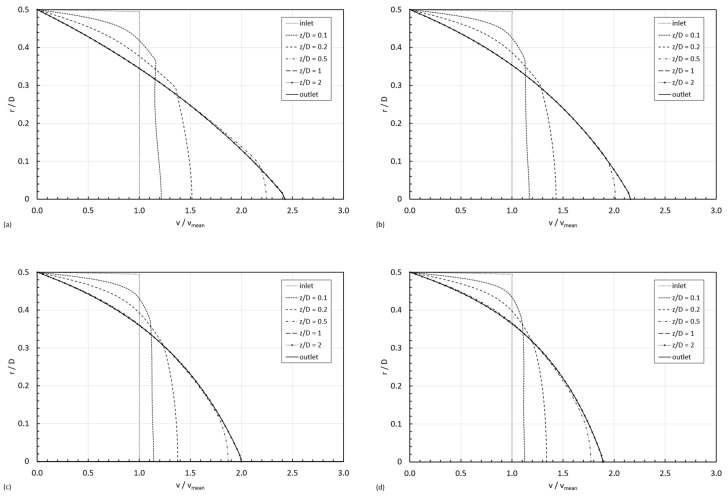
Velocity profiles for the STF 4 formulation in a D=0.1 mm pipe: $v_{in}=$ (**a**) 0.1 mm/s, (**b**) 0.15 mm/s, (**c**) 0.2 mm/s, and (**d**) 0.25 mm/s.

The remaining velocity profiles for the additional inlet velocities are shown in Figure A1, Figure A2, Figure A3 and Figure A4.

**Figure 14:**
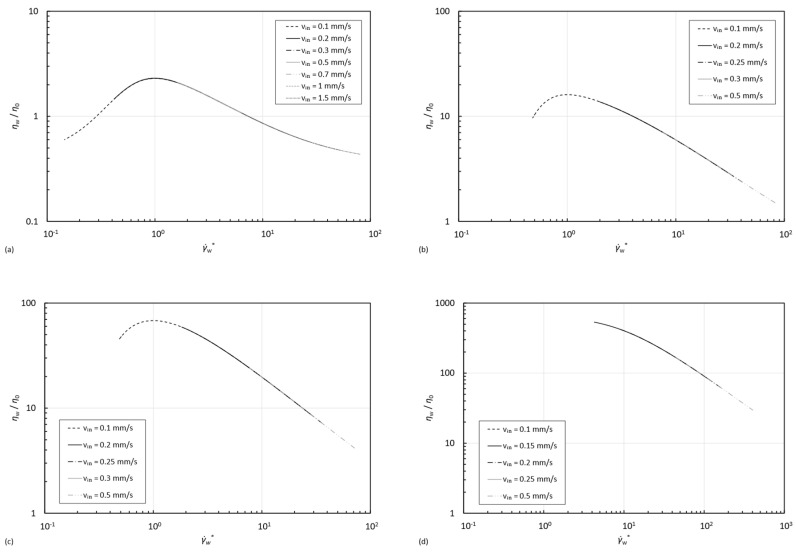
Non-dimensional viscosity versus normalized shear rate along the wall for the formulations: (**a**) STF 1, (**b**) STF 2, (**c**) STF 3, and (**d**) STF 4.

In this study, the range of inlet velocities was selected in such a way that the shear rate on the wall covered the shear thickening region of the viscosity curve of the fluid. In order to better perceive which region of the viscosity curve the flow is encompassing, the evolution of the non-dimensional viscosity, $\frac{\eta }{\eta_{0}}$ with the normalized shear rate along the wall is calculated by Equation (9) and presented in Figure 14.

(9) $${\dot{\gamma }}_{w}^{*}=\frac{\left({\dot{\gamma }}_{w}-{\dot{\gamma }}_{min}\right)}{\left({\dot{\gamma }}_{max}-{\dot{\gamma }}_{min}\right)},$$

Figure 14 allows us to explain the results in Figure 10, as it evidences that only for the STF 1 formulation, the selected velocities ensure that, on the pipe wall, the whole range of the shear thickening region is covered throughout the simulations, as the onset of the shear thickening behavior is clearly visible. The inlet velocities chosen for the remaining formulations resulted in viscosity values past the critical shear rate of the viscosity curve, already reaching the maximum viscosity value and covering mainly the second shear thinning region of the curve; it is even noticeable that for the STF 4 formulation, the peak viscosity on the wall is never achieved since it occurs for a normalized wall shear rate value of 1, and the results clearly indicate that we are in the descendent part of the viscosity curve, corresponding to the second shear thinning region.

### 3.4. Dissipated Power

**Figure 15:**
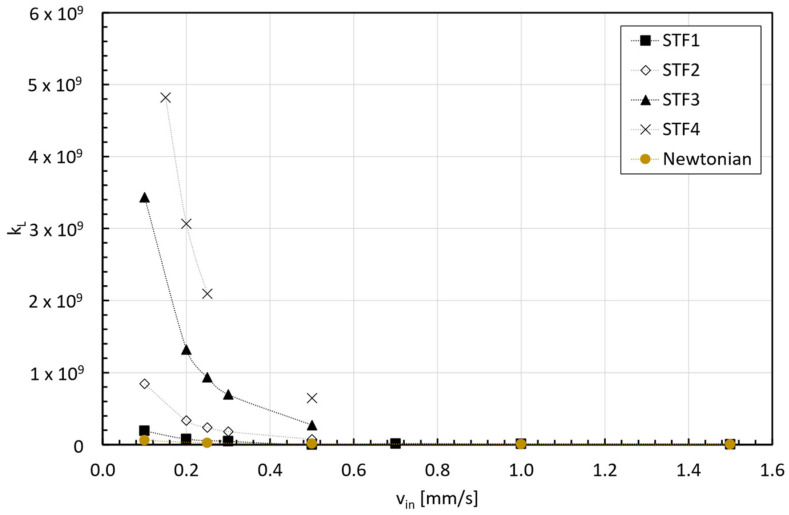
Loss coefficient at coordinate $z=L_{e}$.

It is well known for Laminar flow and Newtonian fluids that the flow pattern within the entry flow region is responsible for a pressure loss that depends on the entrance geometry, and each geometry has an associated loss coefficient due to viscous dissipation (Equation (10)):

(10) $$k_{L}=\frac{\bar{\Delta P}}{\frac{1}{2}\cdot \rho \cdot v_{in}^{2}},$$

being $\bar{\Delta P}=\bar{P}\left(z=0\right)-\bar{P}\left(z=L_{e}\right)$ the average pressure drop within the entry region. The evolution of the loss coefficient with the inlet velocity at the entry length coordinate is illustrated in Figure 15.

**Figure 16:**
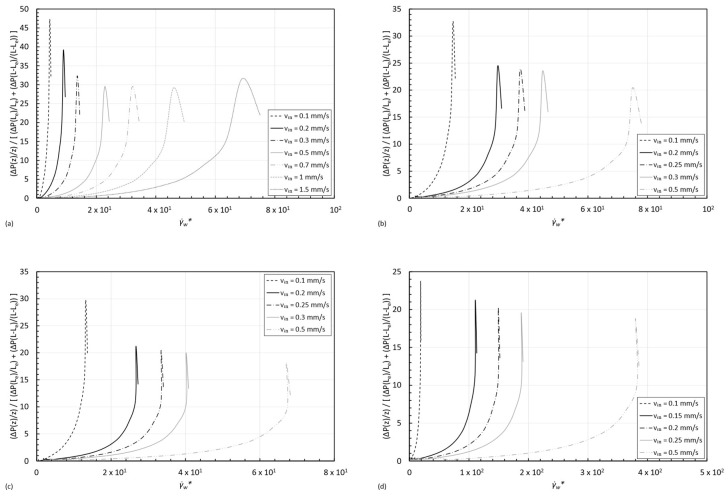
Normalized pressure drop evolution in the entry region ($0\leq z\leq L_{e}$) for the four formulations of four shear thickening fluids with increasing concentration for increasing inlet velocities (**a**) STF 1, (**b**) STF 2, (**c**) STF 3, and (**d**) STF 4.

To observe how the three-region viscosity profile affects the pressure drop in the entry region of the channel ($0\leq z\leq L_{e}$), the evolution of the normalized radial average pressure drop per unit of length ($\frac{\bar{P}\left(r\right)}{z}$) with the normalized shear rate along the wall (${\dot{\gamma }}_{w}^{*}$), calculated by Equation (9) for the four concentrations of STF for increasing inlet velocities is exhibited in Figure 16. These results are consistent with the ones observed in Figure 14: for each inlet velocity, the normalized shear rate along the wall increases and moves from Region I to II, leading to an increase in the viscosity of the fluid and, consequently, an increase in the pressure drop until it reaches the saturation point. After this maximum, the fluid behavior enters Region III of the viscosity curve, and the viscosity diminishes past its maximum value, leading to decreasing values of pressure drop. As it can be observed in Figure 14, the selected inlet velocities for the simulations return viscosity values past the maximum viscosity value of the viscosity curve in almost every case, which explains the decreasing tendency of the normalized pressure drop peak with increasing velocities. These results are of great interest regarding the optimization of microfluidic patterns in applications where STF are used since they can be designed accordingly to control the pressure drop in the fluid flow [27,28,51,52,53] to achieve the desired flow.

**Figure 17:**
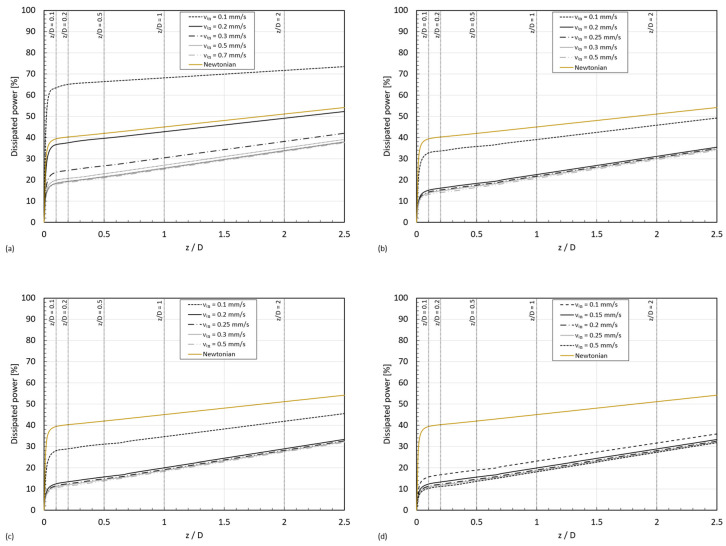
Percentage of dissipated power along the tube in a D=0.1 mm pipe for the formulations: (**a**) STF 1; (**b**) STF 2; (**c**) STF 3; (**d**) STF 4.

As discussed before, in the case of shear-thickening fluids, for a given geometry, the inlet velocity determines the thickening state of the fluid and, consequently, it will affect the pressure loss within the entry region. Figure 17 illustrates the percentage of dissipated power along the tube, calculated by dividing the average of the radial pressure drop along the pipe ($\bar{\Delta P}=\bar{P}\left(r,z=0\right)-\bar{P}\left(r,z\right)$) by the radial average of the total pressure (static pressure, $\bar{P}\left(r,\;z=0\right)$, plus dynamic pressure, ($\frac{1}{2}\cdot \rho \cdot v_{in}^{2}$) (Equation (11)), for the four STF formulations, as well as for a Newtonian fluid:

(11) $$\%\frac{Dissipated\;power}{Input\;power}=\frac{\bar{P}\left(z=0\right)-\bar{P}\left(z\right)}{\bar{P}\left(z=0\right)+\frac{1}{2}\cdot \rho \cdot v_{in}^{2}}\cdot 100,$$

It can be observed that the amount of dissipated power grows nonlinearly within the entry region ($z<L_{e}$).

**Figure 18:**
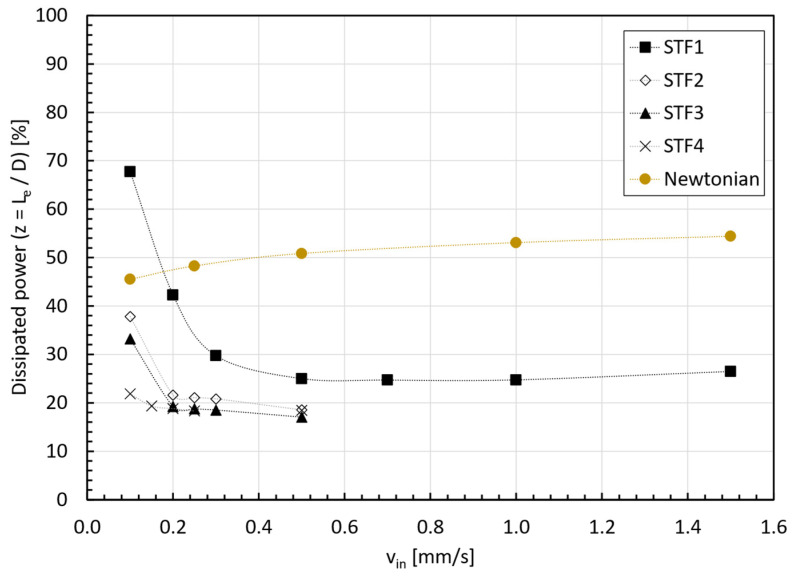
Percentage of dissipated power at coordinate $z=L_{e}$.

The percentage of dissipated power at the entry length coordinate for each STF and the Newtonian case can be observed in Figure 18. Whereas for the Newtonian fluid, the value remains fairly constant, i.e., independent of the inlet velocity for a given geometry; as expected, the percentage of dissipated power at the entry length for the STFs depends both on the inlet velocity and the viscosity curve of the fluid. In all cases, the percentage of energy dissipated decreases with the increase in the inlet velocity until reaching a saturating value; this result can be explained by results shown in Figure 14, where we could observe the transition towards the second shear thinning region in the viscosity curve with the increase in the inlet velocity.

## 4. Conclusions

A systematic and detailed numerical investigation of the entry region effects in the creeping flow regime of shear thickening fluids in 2D axisymmetric microtube flow was conducted in this work. The GNF constitutive model proposed by Khandavalli, Lee, Pasquali and Rothstein [41] described the three regions of the viscosity curve characteristic of STFs exhibiting CST behavior, allowing to analyze the influence of the non-monotonic shape of the viscosity curve with the shear rate on the entrance length. Due to the small characteristic length and the range of velocities of interest, the flow regime analysis laid in the creeping flow regime for very low Reynolds numbers (${Re}_{MR}<10^{-2}$). Therefore, as expected, the entry length was independent of the Reynolds number, a result that is consistent with the literature for power-law fluids [36]; however, the entry length ($\frac{L_{e}}{D}\sim 0.91$) was the same independently from the formulation of the shear thickening fluid considered, i.e., independent from the slope of the viscosity curve within the thickening region (n-index for a power-law fluid). At first sight, this result may seem unexpected and shocking, but if we analyze the stress distribution within the entry region for the four fluids considered, we can observe that there is a maximum in the axial velocity profile at the centerline occurring at the location in which the critical shear stress was minimum. That minimum shear stress value was practically the same for the four fluids, as it is expected from having the same particle, particle size, carrier fluid, similar dispersion quality, but different concentration. Moreover, the minimum in the critical shear stress took place exactly when the extensional flow disappeared. Quantitatively, the value of the peak in the velocity centerline depends on the inlet velocity imposed and the rheology of the fluid. By representing the evolution of the non-dimensional viscosity ($\frac{\eta_{w}}{\eta_{0}}$) with the normalized shear rate along the wall (${\dot{\gamma }}_{w}^{*}=\frac{\left({\dot{\gamma }}_{w}-{\dot{\gamma }}_{min}\right)}{\left({\dot{\gamma }}_{max}-{\dot{\gamma }}_{min}\right)}$) it was possible to unveil which region of the viscosity curve was mostly activated for a given inlet velocity. Thanks to Khandavalli, Lee, Pasquali and Rothstein [41] model, which allowed to cover the three characteristic regions of the CST behavior, it was possible to analyze for the very first time the richness of possible cases in the radial distribution of the viscosity, which depends on the Interplay between the Inlet velocity, the size of the tube, the rheological properties of the fluid, and the location in z-direction, i.e., inside or outside the entry region. The analysis of pressure evolution within the pipe’s entry region confirmed it as the critical area for pressure drop. This finding aligns with experimental results, such as those by Galindo-Rosales [19], where the composite shin guard with the highest impact absorption capacity featured shorter microchannels in its channel geometry design. Moreover, it was also reported for the very first time that the local energy loss due to the entry flow for CST fluids depends nonlinearly on the inlet velocity and also on the viscosity curve of the fluid.

Despite the novelty and practical interest of the results and conclusions withdrawn in this work, this study is, however, limited to shear thickening fluids exhibiting inelastic continuous shear thickening behaviors. Nevertheless, it is well known that these fluids, apart from exhibiting a non-linear relationship between stress and shear rate, also exhibit viscoelastic behavior [54]; nevertheless, the literature lacks a constitutive model able to predict both features simultaneously. The rheological community should work to fill the empty gap in the state-of-art in order to obtain meaningful numerical results, as it has been well documented the importance of shear-induced and extension-induced elastic stresses in energy dissipation for viscoelastic fluids undergoing complex flows [49] and at small length scales [55].

## Figures and Tables

**Table 1:** Fitting parameters of the shear thickening fluid model given in Equation (5) for several nanoparticle concentrations. Reprinted from [41], with permission from Elsevier.

Silica [wt%]	$\eta_{0}$ [Pa∙s]	$\eta_{\infty }$ [Pa∙s]	$B_{1}$	$n_{1}$	$\lambda_{1}$ [ms]	$B_{2}$	$n_{2}$	$\lambda_{2}$ [ms]	A
7.5	3.0	1.0	0.5	0.01	100	3.3	−2.0	62	16
10	3.0	1.0	0.6	0.11	100	10	−3.0	180	48
15	3.3	1.0	1.0	0.15	300	11	−0.9	200	300
20	8.5	1.0	1.0	0.15	300	11	−0.9	300	260

**Table 2:** Mesh characteristics.

Mesh	L Divisions	R Divisions	Number of Elements
1	2000	100	200,000
2	1000	100	100,000
3	1000	50	50,000

**Table 3:** Discretization error calculations.

	$\phi =\frac{v_{c}}{v_{in}}$				
	$\frac{z}{D}$ = 0.1	$\frac{z}{D}$ = 0.2	$\frac{z}{D}$ = 0.5	$\frac{z}{D}$ = 1	$\frac{z}{D}$ = 2
$N_{1},\;N_{2},\;N_{3}$	200,000, 100,000, 50,000				
$r_{21}$	1.30				
$r_{32}$	1.14				
$\phi_{1}$	1.1224	1.3358	1.7682	1.8866	1.8863
$\phi_{2}$	1.1436	1.3900	1.8402	1.8893	1.8906
$\phi_{3}$	1.1444	1.3922	1.8425	1.8886	1.88891
p	7.70	7.38	7.96	3.92	2.74
$\phi_{ext}^{21}$	1.1191	1.3264	1.7577	1.8851	1.8821
$e_{a}^{21}$	1.9%	4.1%	4.1%	0.1%	0.2%
$e_{ext}^{21}$	0.3%	0.7%	0.6%	0.1%	0.2%
${GCI}_{fine}^{21}$	0.4%	0.9%	0.7%	0.1%	0.3%

## Data Availability

Datasets are available upon request from the authors.

## Appendix A

Velocity profiles for the different STF formulations at different inlet velocities: Figure A1 for the STF 1, Figure A2 for the STF 2, Figure A3 for the STF 3, and Figure A4 for the STF 4 (Table 2).
